# Prosthetic knee joint infection caused by α-hemolytic *Streptococcus* species: a case report

**DOI:** 10.1186/s13256-023-03905-1

**Published:** 2023-07-26

**Authors:** Masoud Mardani, Jafar Mohammadshahi, Roghayeh Teimourpour

**Affiliations:** 1 Infectious Diseases and Tropical Medicine Research Center, Shahid Beheshti University of Medical Sciences, Tehran, Iran; 2 Department of Infectious Diseases, School of Medicine, Ardabil University of Medical Science, Ardabil, Iran; 3 Department of Microbiology, school of medicine, Ardabil University of Medical Science, Ardabil, Iran

**Keywords:** *Staphylococcus *species, Knee, Arthroplasty, Infection, Case report

## Abstract

**Background:**

Knee arthroplasty is an orthopedic surgical procedure in which a damaged joint is replaced with an artificial one. It is estimated that 1–2% of knee arthroplasties will encounter infection over their lifetime. Although α-hemolytic *Streptococcus* species play an important role in prosthetic joint infection, they are less common than staphylococcal species.

**Case presentation:**

In this report, a 50-year-old Iranian woman was diagnosed with prosthetic knee joint infection based on clinical, radiological, and laboratory findings. She was diabetic and had undergone a left total knee arthroplasty, which, 18 months after the surgery, presented pain, erythema, and edema in that knee. The primary culture of knee aspirate was positive for α-hemolytic *Streptococcus *species, but following antibiotic medication, culture was negative. The primary antibiotic regime was vancomycin and meropenem, which was changed to cefepime for the management of the infection based on the results of antimicrobial susceptibility testing.

**Conclusions:**

This report indicated the clinical presentation and management of the patient with prosthetic joint infection in which the patient recovered without any severe complications or surgical intervention.

## Background

Prosthetic joint infection (PJI) is an infection of the joint prosthesis and adjacent tissue. It is estimated that about 1–2% of people with prosthetic hip or knee replacement encounter an infection. In this regard, early diagnosis and appropriate management are essential to ease pain, restore joint function, and prevent more complications [1]. Epidemiological studies over the past decades have indicated that, with the increasing number of hip and knee arthroplasties, the rate of PJI has been increasing in parallel [2]. Obesity, diabetes mellitus, rheumatoid arthritis, malnutrition, smoking, immunosuppression, and age over 50 years are the most common risk factors for developing PJI [3, 4]. Several factors, including the virulence of the organism, host immune responses, the structure of soft tissue surrounding the joint, and the route of primary infection, can affect the clinical presentation of PJI. In general, local pain, fever, joint swelling, and erythema or tenderness are the most common clinical symptoms. For laboratory investigations, peripheral blood for verifying blood index and synovial fluid for culture should be taken from the patient. Leukocytosis, the elevation of erythrocyte sedimentation rate (ESR), and C-reactive protein (CRP) in a blood sample as well as the positive synovial fluid culture in the presence of a high white blood cell (WBC) count in the synovial fluid examination are the main laboratory findings in primary PJI diagnosis. Based on the recommendation from the Infectious Diseases Society of America (IDSA), if the isolated bacterium is *Staphylococcus*, intravenous antibiotic therapy and then an oral antibiotic medication should be prescribed for 2–6 weeks and 3 months, respectively, for complete eradication of the microorganism; for other bacteria, 4–6 weeks of antimicrobial therapy should be indicated [5]. The present study aimed to report a rare prosthetic knee joint infection caused by α-hemolytic *Streptococcus* and demonstrate the successful management of the infection.

## Case presentation

**Fig. 1:**
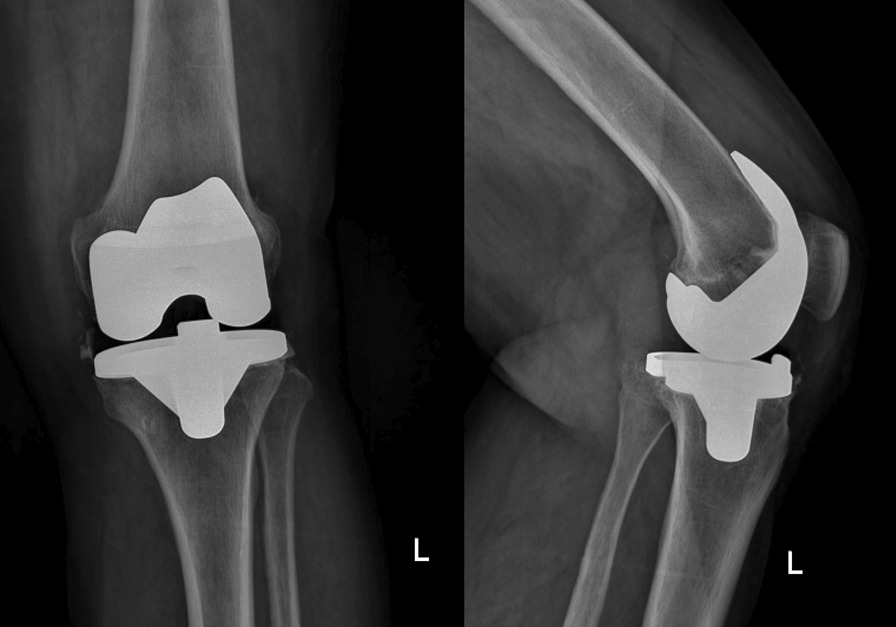
Radiographic image of the implement in the left knee

A 50-year-old Iranian housewife with a total replacement of the left knee joint (Fig. 1) presented to our department. On the primary examination, fever (38 °C), blood pressure (BP) of 125/75 mmHg, pulse rate (PR) of 75 beats per minute, and respiratory rate (RR) of 15 breaths per minute, along with chilling, nausea, swelling, and pain in the left knee that had started 3 days ago, were recorded. Except for decreased ability to move the joint, other predominant physical and neurological symptoms were not observed.

In the past medical history (PMH), the patient had a history of diabetes mellitus with insulin therapy and a left knee joint replacement 1.5 years ago. Except for these cases, she had no other significant past medical history or notable family or hereditary history of specific disease and medication use. She had given birth to three children and denied alcohol consumption and smoking. For controlling diabetes, she used neutral protamine Hagedorn (NPH) insulin [20 units every 12 hours, subcutaneous injection (SQ)] and regular insulin (12 units in the morning and 8 units at night, SQ).

For diagnosis, a peripheral blood sample and aspirated synovial fluid were taken and were subjected to biochemical, hematological, and microbiological testing.

A peripheral blood sample of 10 ml was obtained and inoculated into a blood culture bottle aseptically; it was negative after a 72-hour period of incubation at 37 °C. A synovial aspiration sample of 2 ml was taken from the patient and was dropped into collection tubes containing liquid ethylenediaminetertraacetic acid (EDTA) aseptically. The specimen was cultured into selective media including blood agar (for isolating aerobic bacteria), chocolate agar (incubated in a candle jar for isolating aerobic capnophilic bacteria), thioglycollate broth (for recovering anaerobic bacteria), eosin methylene blue agar (EMB; for recovering gram-negative bacteria), and subro dextrose agar (for isolating fungi). Also, two sets of slides were prepared and stained with gram and acid-fast staining. Polymerase chain reaction (PCR) and acid-fast staining were employed for identification of acid-fast bacilli, which were negative. Susceptibility to sulfamethoxazole-trimethoprim (SXT), neomycin, and bacitracin discs was used for the differentiation of α**-**hemolytic *Streptococcus* species (spp.) from other *Streptococcus* spp.

Lab biomarkers of blood and synovial specimens at the time of admission are presented in Table 1. In synovial cell counting examination, many WBC in which neutrophils accounted for 80% of whole cells were reported.

**Table 1:** Laboratory findings of patient on hospital admission

Lab study type	Variable	Lab value	Reference range
Blood	WBC	12.6 (× 10^3^ per μl)	4.5–11 (× 10^3^ per μl)
	Hb	11.4	14–16.5 g/dl
	Blood sugar	224	70–140 mg/dl
	Urea	38	10.6–48.5 mg/dl
	Cr	1	0.7–1.4 mg/dl
	ESR	80	0–30 mm**/**hour
	CRP	278	0–10 mg/l
	PLT	172	150–440(× 10^3^ per μl)
Synovial fluid	WBC	many	MN = 10%, PMN = 90%
	RBC	1500 cell/ml	

*Hb* hemoglobin, *WBC* white blood cell, *ESR* erythrocyte sedimentation rate, *CRP* Creactive protein, *Cr* creatinine, *RBC* Red blood cell, *PLT* platelet cell, *MN* mononuclear, *PMN* polymorphonuclear

At the time of admission, since the causative agent was unknown, on the first day, empirical therapy including vancomycin (1 g every 12 hours, intravenous injection for 2 days) and meropenem (1 g every 8 hours, intravenous injection for 2 days) were initiated to target suspicious invasive agents like *Staphylococcus* spp. and gram-negative bacilli. On the second day, edema, warmness, and redness declined but fever remained. The results of the blood and synovial culture were revealed on the third day. Only the α-hemolytic *Streptococcus* spp. was isolated from the synovial specimen, which was subjected to antibiogram testing. Based on the results of antimicrobial susceptibility testing on the third day, antibiotic medication was changed to cefepime (2 g every 8 hours, intravenous injection) for 14 days. Transthoracic echocardiogram (TTE) and transesophageal echocardiogram (TEE) tests were recommended by an infectious disease (ID) specialist and were performed by an echocardiography cardiologist, who indicated that there was no sign of endocarditis. In orthopedic consultation, according to radiographic results, the exchange of removable components of the implant was not recommended; the antibiotic therapy was continued. The patient was treated with intravenous antibiotics for 14 days and was discharged after partial recovery. At the time of discharge, the patient's symptoms were entirely resolved, there was no movement disorder, and there was only slight swelling in her left knee. Oral medication with cephalexin was prescribed for her for 2 weeks. One month after discharge, the patient did not have any specific complaints and joint restrictions, and the test results of erythrocyte sedimentation rate (ESR) of 25 and C-reactive protein (CRP) of 8 were in normal range.

## Discussion and conclusions

Arthroplasty is an orthopedic surgical procedure in which a musculoskeletal joint damaged due to trauma or arthritis is replaced to relieve pain and restore the joint's function. The incidence rate of postoperative knee joint replacement is meager and is approximately 0.01–3.4% [6]. The onset of PJI is divided into three stages: early, delayed, or late, which initiate at 3 months, 12 months, and > 12–24 months after surgery, respectively. Generally, the virulence of the microorganism and the mode of infection determine the onset time of the disease. Late-onset PJI is usually related to a less virulent microorganism and hematogenous spread of infection in comparison to early and delayed PJI, which are mostly due to hypervirulent microorganisms and intraoperative contamination. In most cases, PJI occurs due to contamination during operation. Colonization of microorganisms on the implant's surface can occur through the direct contamination or hematogenous spread of infection from distant sites. Previous studies have shown that *Staphylococcus *spp. account for 50–60% of total PJI infections, while only 10% of cases were attributed to *Streptococcus* spp. [3]. For diagnosis of PJI, a combination of clinical, laboratory, radiological, and pathological results should be noted. However, none of them has 100% accuracy for diagnosis of PJI. According to different studies, imaging and synovial fluid analysis have the highest sensitivity and specificity [7–10]. In the case of clinical findings, the presence of a sinus tract is a major criterion for PJI diagnosis [11], but our patient had no sinus tract. Based on previous reports, the mortality rate of PJI is about 2–4%; however, in some cases, such as involvement with *Staphylococcus aureus*, mortality rate can reach 7%, which reflects the high virulence of this microorganism [12, 13].

In the management of PJI, elimination of the infection, restoring the function of the infected joint, and minimizing PJI-related morbidity and mortality should be considered. In general, for these patients, surgical intervention and antibiotic therapy should be noticed [14, 15]. In this study, surgical intervention and implementation exchange were not done, and the patient only received intravenous antibiotics.

This case report presented the clinical manifestation and pharmacological management of a prosthetic joint infection due to α-hemolytic *Streptococcus* spp. Compared with staphylococci spp., PJIs due to α-hemolytic *Streptococcus* spp. are less common.

α-Hemolytic streptococci spp. are a group of 50 species found to be a part of the human saliva microbiome. They can invade the blood following dental manipulation and trauma and cause a wide range of infections, so they are one of the most important causes of endocarditis and brain abscesses. PJIs can occur due to hematogenous spread of bacteria or surgical site infections by commensals. Most hematogenous PJIs arising with α-hemolytic streptococci occur months or years after arthroplasty [16].

In a retrospective multicenter cohort study, prosthetic joint infections (PJIs) with *Streptococcus* spp. were investigated from 2001 to 2009. Most of the conditions (57.9%) were treated using surgical debridement and antibiotic therapy. In this study rifampicin and levofloxacin in combination were found to be the most effective antibiotics against *Streptococcus* spp. The highest rate of comorbidities was related to diabetes mellitus (35.6%) and malnutrition (28.7), respectively. This study concluded that the outcomes of streptococcal PJIs may not be as good as reported in previous studies [17]. In line with this study, our case was a diabetic patient, but her response to antibiotic therapy was satisfactory.

Marongiu reported a case in which a 72-year-old male had a late onset of PJIs due to *Streptococcus anginosus*. The infection was treated through two-stage revision arthroplasty and postoperative antibiotic therapy (vancomycin and levofloxacin) [18]. Several previous studies regarding streptococcal prosthetic joint infections (PJIs) are reviewed and summarized in Table 2. According to these reports, the antibiotic regimen used in our study differs from previous similar studies, which could be due to the difference in regional antibiotic resistance patterns and the virulence of the involved streptococcal strains.

**Table 2:** Summary of some previous streptococcal prosthetic joint infections

	Type of study	Age	Reported comorbidity	The most effective antibiotics	Year
Fiaux et al. [17]	Retrospective	69.1 ± 13.7	Diabetes mellitus 35.6%, rheumatoid polyarthritis 9.1%, chronic renal disease 14.9%, chronic liver disease 10.3%, malnutrition 28.7%, neoplasia 8%, corticosteroids 12.6%	Rifampicin-levofloxacin	2001–2009
Marongiu [18]	Case report	72	Hypertension, urinary tract infection	Vancomycin, levofloxacin	2019
Huotari [23]	Retrospective	66.4 ± 12.3	Rheumatoid arthritis, immunosuppressive medication, renal failure	Amoxicillin, clindamycin, and cephalexin; rifampin	2008–2017
Tamayo [24]	Retrospective	Mean age: 72	Malignancy	β-Lactams, rifampin	2003–2012
Mahieu [25]	Retrospective	77	Diabetes, chronic arterial hypertension, chronic heart failure, chronic obstructive pulmonary disease, alcohol abuse, rheumatoid arthritis, chronic renal failure, active cancer, cirrhosis	Amoxicillin, levofloxacin, rifampicin	2010–2012
Akgün [26]	Retrospective	71	–	Rifampin, penicillin, levofloxacin, clindamycin, co-trimoxazole	2009–2015
Lam [27]	Retrospective	70	–	Amoxicillin, penicillin V, clindamycin, rifampicin	2011–2015
Balato [6]	Case report	22	Obesity	Piperacillin/tazobactam**,** amoxicillin, clindamycin	2019
Olson [28]	Case report	92	Dental procedure	Piperacillin-tazobactam**,** vancomycin	2019

Also, according to previous the studies, anaerobic bacteria play an important role in causing delayed and late PJIs. Isolation of these bacteria from clinical samples is difficult and requires enriched medium- and long-term incubation of cultures (4–6 days), so the probability of false negative results in their identification is high. The use of molecular methods and enriched culture media will facilitate their isolation and identification. In this case, due to the limitation in the detection of anaerobic bacteria, the role of anaerobes in causing infection cannot be completely ignored [19, 20].

In conclusion, joint arthroplasty has enhanced the lives of millions of people worldwide. Postoperative infection occurs rarely, but appropriate management of such a condition would significantly decrease the mortality rate and severe complications. Here, we report a rare case of PJI in a older woman with diabetes without any other predisposing factors for joint infection. Among the different types of bacterial agents that are involved in PJI, α-hemolytic *Streptococcus* spp. are less frequent; despite this, α-hemolytic *Streptococcus* spp. are an important part of commensal oral flora and can easily spread to the blood and seed at the surgical site. Diagnosis based on culture examination of aspirated synovial fluid and administration of effective antibiotics based on antimicrobial susceptibility testing plays an important role in the management of such infection. Some previous reports indicated that antibiotic treatment outcomes were comparable to the open surgery procedure [21, 22]. As presented in this case, our patient recovered from infection without surgical intervention following effective antibiotics medication.

## Data Availability

Not applicable.

## Abbreviations

PJI: Prosthetic joint infection
CRP: C-reactive protein
WBC: White blood cell
IDSA: Infectious Diseases Society of America
BP: Blood pressure
PR: Pulse rate
RR: Respiratory rate
PMH: Past medical history
NPH: Neutral protamine Hagedorn
SQ: Subcutaneous injection
EDTA: Ethylenediaminetertraacetic acid
EMB: Eosin methylene blue agar
PCR: Polymerase chain reaction
TTE: Transthoracic echocardiogram
TEE: Transesophageal echocardiogram
ID: Infectious disease
ESR: Erythrocyte sedimentation rate
Hb: Hemoglobin
Cr: Creatinine
PLT: Platelet count
RBC: Red blood cell
